# Repurposing Estrogen Receptor Antagonists for the Treatment of Infectious Disease

**DOI:** 10.1128/mBio.02272-18

**Published:** 2018-12-18

**Authors:** Marhiah C. Montoya, Damian J. Krysan

**Affiliations:** a Clinical and Translational Science Institute, University of Rochester School of Medicine and Dentistry, Rochester, New York, USA; b Department of Pediatrics, Carver College of Medicine, University of Iowa, Iowa City, Iowa, USA; c Department of Microbiology/Immunology, Carver College of Medicine, University of Iowa, Iowa City, Iowa, USA; University of Texas Health Science Center at Houston

**Keywords:** anti-infective, estrogen receptor antagonists, repurposing

## Abstract

The concept of repurposing previously approved medications to the treatment of new indications by taking advantage of off-target effects has gained traction in recent years, particularly in areas of medicine that do not offer large profits to pharmaceutical firms. As infectious disease discovery research has declined among large pharmaceutical companies, the potential payoff of repurposing has become attractive. From these efforts, the triphenylethylene class of selective estrogen receptor modulators related to tamoxifen has shown activity against a wide range of medically important human pathogens, including bacteria, fungi, parasites, and viruses. Because it has activity against many pathogens affecting people in resource-limited areas of the world, TAM and related drugs may be particularly useful. Here, we review the *in vitro*, *in vivo*, and mechanistic studies of the anti-infective activity of tamoxifen, toremifene, clomiphene, and their analogs. We also discuss the pharmacologic properties of this privileged scaffold and its potential utility in treating infectious diseases.

## Abstract (precis)

The concept of repurposing previously approved medications to the treatment of new indications by taking advantage of off-target effects has gained traction in recent years, particularly in areas of medicine that do not offer large profits to pharmaceutical firms. As infectious disease discovery research has declined among large pharmaceutical companies, the potential payoff of repurposing has become attractive.

## INTRODUCTION

Drug repurposing is the application of a molecule that has been clinically approved to treat a human medical condition to the treatment of another medical condition for which it was not previously indicated. In the literature this drug discovery concept has also been termed drug repositioning, rediscovery, and reprofiling in addition to others (1). Drug repurposing can expedite the transition of a new therapy from bench to bedside because existing pharmacologic and toxicologic data can be applied to the new indication, thereby shortening the drug development timeline (2). Utilizing existing data generated during the initial drug development provides an advantage over novel drug development and accelerates the processes required to bring a drug to clinic compared to developing a new molecule that has not been approved by governing bodies like the United States Food and Drug Administration. Well-known repurposed drugs include sildenafil, bimatoprost, and the infamous thalidomide. Thalidomide is an interesting example of drug repurposing due to its resurrection through drug repurposing. Thalidomide was initially developed as a sedative and antinausea medication during pregnancy and tragically caused devastating birth defects. Many years later, its anti-inflammatory properties were discovered and it was repurposed to treat erythema nodosum leprosum, a condition associated with leprosy, as well as multiple myeloma (2, 3). The case of thalidomide illustrates how previous studies generated as part of the initial development and the data gathered after clinical approval provided a solid foundation for its repurposing to a new and important indication.

Drug repurposing provides a particularly attractive approach to address unmet clinical needs in the area of infectious diseases. The current economics of pharmaceutical drug development are such that few large pharmaceutical concerns have active discovery programs for anti-infectives. In addition, many of the most pressing unmet clinical needs exist in resource-limited regions that do not offer lucrative markets for novel drug discovery or development (4). Toward this end, many screening assays have been applied to collections of FDA-approved drugs either as dedicated repurposing campaigns or as proof-of-principle screens. From these various screens, specific molecules or drugs repeatedly emerge as hits, indicating they have diverse biological activity and effects (5). Although some of these molecules are nuisance molecules that have nonspecific activities and present as false-positive hits in high-throughput drug screens, such as the so-called pan-assay interference (PAIN) class molecules, others represent examples of privileged scaffolds that have useful activity (5 – 8). The precise definition of a privileged scaffold has been debated in the literature; the two most common definitions are (i) a single molecule that binds or interacts with multiple targets and (ii) multiple molecules with the same molecular scaffold that are biologically active (6). Regardless of the definition, the concept of privileged scaffolds has been used to generate focused libraries that can be used to optimize the chemical profiles for specific applications (6, 7, 9, 10). For example, quinoline is a privileged scaffold that comprises the backbone for agents such as quinine (antimalarial), camptothecin (anticancer), and broxyquinoline (antiseptic) (6).

**FIG 1:**
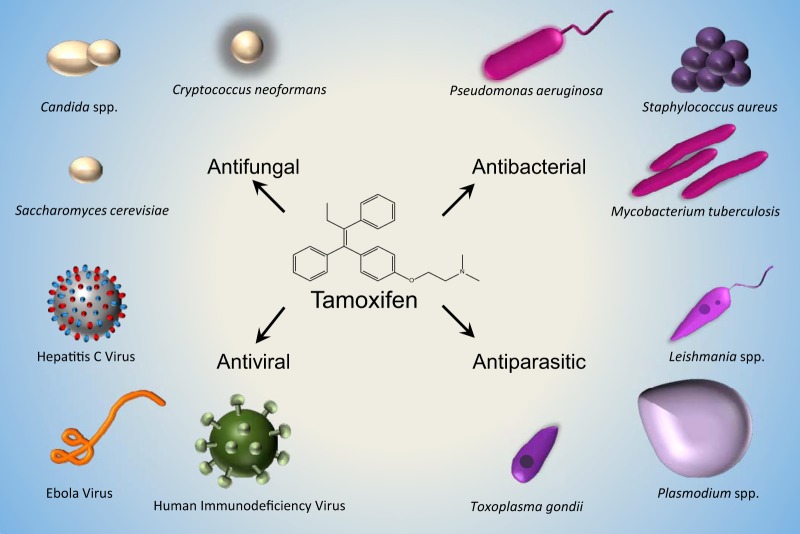
The scope of microorganisms against which tamoxifen and its analogs are active. Microorganisms are not depicted to scale.

This review will discuss the triphenylethylene scaffold as a privileged scaffold, specifically focusing on the infectious disease applications of the selective estrogen receptor modulators (SERMs) tamoxifen (TAM), toremifene (TOR), and clomiphene/clomifene (CLM), including a discussion of the various mechanisms of action and targets that mediate these non-estrogen receptor activities (Fig. 1).

### Triphenylethylene as a privileged scaffold.

The triphenylethylene class of molecules represents the backbone of TAM and other TAM-related estrogen receptor antagonists, including TOR and CLM. TAM, TOR, and CLM are best known for their activity as SERMs. TAM was initially discovered and studied as a potential contraceptive, but it proved ineffective for that purpose (11). Ultimately, it was investigated for its potential as an anti-breast cancer drug (11). TAM competitively binds to estrogen receptors in breast cancer cells and, thereby, inhibits an important proliferative signal (12 – 14). Currently, it is most commonly used as a maintenance agent in patients with estrogen-receptor-positive tumors. Additional conditions that respond to the estrogen receptor antagonist properties of triphenylethylene-based SERMs include osteoporosis, hypercholesterolemia, and gynecomastia (14). Supporting the privileged nature of the triphenylethylene scaffold, tamoxifen has been repurposed to treat estrogen-receptor-independent conditions such as glioblastoma multiforme, desmoid tumors, and bipolar disorder; although the exact mechanism of these activities is not known, the ability of TAM to inhibit protein kinase C appears to play a role in their activity (15 – 17). As discussed in previous reviews, a variety of other targets have been proposed to mediate the various effects of tamoxifen and its analogs (18). Here, we review literature on the triphenylethylenes TAM, TOR, and CLM as repurposed molecules for infectious disease.

### Antifungal activity.

The antifungal activity of TAM against Saccharomyces cerevisiae, Candida albicans, and Cryptococcus neoformans was reported in the late 1980s, early 1990s, and early 2000s, respectively (19 – 21). We will focus on the activity of the triphenylethylenes against C. albicans and C. neoformans. *In vitro* time-kill experiments of log-phase C. albicans cells showed TAM at 7.5 µg/ml exhibited antifungal activity, similar to what was shown in earlier S. cerevisiae experiments (19). The antifungal activity of TAM was maintained against both log-phase and stationary-phase yeast cells, with greater effects seen against log-phase cells (22, 23). Additionally, a similar time-kill experiment comparing TAM to miconazole at equal molar concentrations showed identical antifungal activity (24). These initial data were subsequently validated using standard Clinical and Laboratory Standards Institute (CLSI) antifungal *in vitro* susceptibility methods against C. albicans, C. parapsilosis, C. dubliniensis, C. glabrata, and C. tropicalis (21). Using the CLSI M27-2A protocol, TAM showed antifungal MICs ranging from 8 to 64 µg/ml against all listed species. Interestingly, CLM is much less active as an antifungal, with MICs of ≥32 µg/ml for all fungi tested (21). Combination of tamoxifen with other antifungal drugs has shown mixed results against planktonic C. albicans cells with mainly additive affects observed (25) In contrast, TOR combined with either amphotericin B (AmB) or caspofungin led to reduced biofilm formation (26).

Both TAM and TOR penetrate the CNS well, and thus, their activity against Cryptococcus neoformans, the most common cause of fungal meningitis, was of interest. Against C. neoformans var. *grubii*, TAM and TOR have MICs of approximately 8 µg/ml (27). Importantly, the main two TAM metabolites, 4-hydroxytamoxifen and endoxifen, were slightly more active against C. neoformans var. *grubii* with MICs of 4 µg/ml (27). Combination studies using TAM showed synergy with AmB and fluconazole and additivity with flucytosine. Importantly, the combination of TAM or TOR and fluconazole is fungicidal at concentrations achievable in human serum (25, 27).

TAM is active in murine models of disseminated candidiasis and cryptococcosis. In the disseminated candidiasis model, 200 mg/kg of body weight/day TAM for 7 days prior to infection reduced kidney fungal burden by 1.5 log_10_ at 2 days postinfection, indicating that the molecule has *in vivo* activity (21). Although sufficient levels of TAM were not achievable for activity against C. neoformans as a single agent, TAM combined with intermediate levels of fluconazole improved clearance of the fungus from the brain relative to either agent alone (27).

A variety of modes of action and targets have been proposed for the antifungal activity of TAM/TOR. Early mode-of-action studies indicated that TAM inhibited lipid peroxidation; in addition, it was proposed that accumulation of TAM in the membrane may affect membrane fluidity in yeast (28, 29). Consistent with that notion, TAM, TOR, and CLM disrupt yeast cell integrity, resulting in lysis (21, 27). One of the best-characterized off-target effects of tamoxifen and triphenylethylenes is calmodulin, a key calcium binding protein with essential functions in most eukaryotes. Previously, we have shown that tamoxifen directly binds to fungal calmodulin by thermal shift assays, inhibits calmodulin-mediated calcineurin activation, and disrupts nuclear localization of the calcineurin-regulated transcription factor Crz1 (27). In addition, overexpression of the calmodulin gene reduces the susceptibility of strains to tamoxifen while calmodulin loss-of-function mutants are hypersusceptible to the drug (21, 27). Finally, tamoxifen analogs with increased calmodulin antagonism have increased antifungal activity (27, 30). Though there may exist multiple targets, it appears the target that drives a substantial portion of the antifungal activity of TAM and TOR is calmodulin (21, 27). A phase II clinical trial of tamoxifen as an adjuvant to standard-of-care therapy for cryptococcal meningitis is under way (31).

### Antiviral activity.

The studies exploring the antiviral activity of triphenylethylene-based SERMs have mainly focused on three infections: human immunodeficiency virus (HIV), hepatitis C virus (HCV), and Ebola virus (EBOV). TAM is active against HIV, HCV, and herpes simplex virus 1 (HSV-1), while CLM and TOR are active against EBOV.

As part of the initial search for anti-HIV drugs in the early 1990s, TAM was identified as a disruptor of viral replication during chronic infection based on the 4B-phorbol-12-myristate-13-acetate-mediated model and as a disruptor of HIV-associated transactivation in cells of monocytic and T-cell lineages at half-maximal inhibitory concentrations (IC_50_) of ≤10 µM (32). TAM also inhibited HIV replication in nonstimulated, HIV-infected lymphocytes through pathways independent of its antiestrogen activity (33). As with other off-target effects in human cells, the mechanism of action was attributed to inhibition of PKC and interaction with other targets in the NF-κB pathway (32). TAM’s activity was not better than the HIV treatment option available at the time, AZT (33). Thus, these *in vitro* data did not lead to *in vivo* or clinical studies.

TAM (1 µM) inhibits HCV replication by interfering with the association of estrogen receptor alpha with RNA-dependent RNA polymerase NS5B (34). As a result, TAM interferes with the formation of the replication complex and ultimately prevents viral genome replication (34). Further studies have shown that TAM protects cells from HCV-induced cytopathic effects at ≤8 µM and blocks HCV core protein expression if given at or before infection (35). The activity of TAM against both HIV and HCV is increased when cells are exposed to the drug prior to infection. More recent characterization of the activity of SERMS against HCV by Murakami et al. indicates that TAM inhibits viral attachment, entry, replication, and exit (36, 37). Interestingly, this multiple-step inhibitory activity of TAM was also observed in a single study of its activity against HSV-1 replication (38). Specifically, a chloride channel-inhibitory activity of TAM is thought to prevent viral fusion, cell penetration, and translocation (38). Additionally, TAM inhibits viral production in both wild-type and acyclovir-resistant strains (38).

The activity of SERMs against EBOV was discovered through a dedicated repurposing screen of FDA-approved drugs in 2013. Although this screen identified only CLM and TOR, subsequent targeted assays found that TAM, raloxifene (RLX), and the CLM stereoisomers enclomiphene and zuclomiphene are also active against EBOV (39 – 43). CLM and TOR have *in vitro* antiviral activity against a variety of EBOV strains with IC_50_ values ranging from 2.42 to 11.1 µM and 0.162 to 6.17 µM, respectively (41, 44). In a murine EBOV infection model, male and female mice were treated with CLM or TOR at 60 mg/kg/day with dosing on day 0, day 1, and alternating days thereafter for 10 days. At 28 days postinfection, 90% of CLM-treated mice survived (*P < *0.0001) while 50% of TOR-treated mice survived (*P = *0.0441) (41). A follow-up *in vivo* study of CLM using an alternative dosing strategy did not observe a survival benefit, suggesting that a balance between activity and toxicity was quite important for efficacy (42). *In vitro* mechanistic experiments suggest that CLM and TOR inhibit viral entry into the host cell in a dose-dependent manner with specificity to virus-like particles containing EBOV GP1,2 (41). Further studies of the crystal structure suggest that TOR binds in the pocket between GP1 and GP2 and, consequently, may decrease stability of the complex. In this way, TOR may prevent the conformational changes necessary for GP1,2-promoted viral fusion with the endolysosomal membrane (45). Finally, these promising results have led to a series of studies evaluating the combination of TOR/CLM with a variety of other molecules (46 – 48). Since some of these combinations have shown synergistic activity, it will be interesting to see whether more effective therapies based on the SERMs can be developed.

### Antiparasitic activity.

TAM is active against a wide variety of human parasites, including *Leishmania* spp., Toxoplasma gondii, *Plasmodium* spp., Trypanosoma cruzi, and Taenia solium. The most extensively studied antiparasitic activity of TAM is as an antileishmania drug. *In vitro* TAM is active against L. amazonensis promastigotes and amastigotes with IC_50_ values of 16.4 ± 0.2 µM and 11.1 ± 0.2µM, respectively (49). IC_50_ values range from 9.0 to 20.1 µM for L. braziliensis, L. major, L. chagasi, and L. donovani (49). Miguel et al. (50) also found highly consistent activity of TAM against a wide range of clinical isolates from cutaneous and visceral leishmanial infections. TAM EC_50_ concentrations ranged from 2 to 15 µM for promastigotes and amastigotes from L. infantum chagasi, L. braziliensis isolates, and L. amazonensis isolates (50). *In vitro* combination studies to treat cutaneous leishmaniasis with TAM and AmB showed only additive or indifferent effects (51).

Compared to its antifungal activity, the antileishmanial activity of TAM has been studied quite extensively using both cutaneous and visceral mammalian infection models. Using the cutaneous infection model with BALB/c mice and L. amazonensis, Miguel et al. found that daily treatment with TAM (20 mg/kg) for 15 days resulted in a decrease in lesion size, parasite load, and ulcer development (52). The same group extended these findings by examining a mouse model of L. braziliensis cutaneous infection and a hamster model of L. chagasi visceral infection (51). Animals were treated with TAM (20 mg/kg/day) for 15 days (51), resulting in decreased lesion size, decreased parasite burden, and increased survival. Untreated mice and hamsters died within 11 days and 18 days, respectively, while 100% of TAM-treated animals survived (51). Finally, oral administration of TAM (20 mg/kg/day) for 15 days in Swiss albino mice also decreased parasite burden and lesion size of cutaneous L. major infections but did not eradicate the parasite from infecting the wound (53). Overall, TAM has shown promising results in *Leishmania* infection models.

The combination of TAM with other antiparasitic drugs has also been studied using *in vivo* models of infection (54 – 56). Despite the fact that the combination of TAM with AmB had additive/indifferent fractional inhibitory concentration indexes (FICIs) ranging from 0.57 to 1.29 against promastigotes or intracellular amastigotes *in vitro* (54), this combination at the maximal tolerated dose (26 mg/kg/day TAM and 4 mg/kg/day AmB) showed greater reduction in lesion size and parasite burden than each drug alone (54). The combination of TAM with miltefosine has also been studied. *In vitro*, TAM-miltefosine showed FICIs of 1.32 for promastigotes and 0.63 for intracellular amastigotes (55). Combination of TAM and miltefosine in BALB/c mice at one-half the median effective dose (ED_50_) for each drug resulted in a decrease in lesion size and parasite burden compared to each drug along (55). Last, topical TAM and meglumine antimoniate treatment of cutaneous leishmaniasis (L. amazonensis) reduced lesion size and parasite burden (56).

The mechanism of action for the activity of TAM against *Leishmania* has been hypothesized to involve the induction of an altered membrane physiology, or in combination with AmB, TAM may reduce the toxic effects of AmB on the host (54, 55). Additionally, TAM has been shown to induce early and late apoptosis in L. major promastigotes *in vitro* in a dose-dependent manner in L. amazonensis (57, 58).

TAM first showed promise for the treatment of T. gondii infections in 1986. An *in vivo* experiment using a dose of 1.2 µmol TAM daily for 3 days reduced brain cyst formation by approximately 50% in mice; interestingly, estradiol enhanced parasite burden in the same model (59). Based on these observations, the authors suggested that the estrogen-induced alteration of host susceptibility to T. gondii is related to its antiestrogen activity. In contrast to that hypothesis, recent T. gondii *in vitro* studies showed that TAM significantly reduces parasite replication and invasion by interfering with initial contact and adhesion to the host cell (60). The proposed mode of action is that TAM induces xenophagy or autophagic destruction through a mechanism that is independent of estrogen receptor antagonism (60).

For helminth cestode parasite Taenia crassiceps, initial *in vivo* studies concluded that parasite infection was also greatly influenced by the presence of estrogen, a hormone known to increase parasite burden, and treatment with TAM caused a reduction in parasite load (61). Further *in vivo* studies using TAM for treatment of T. crassiceps showed an 80% and 50% reduction in parasite burden in female mice and male mice, respectively, when given TAM (0.5 mg/kg) for 1 week of prophylaxis before infection (total time of 8 weeks of infection and 9 weeks of treatment) (62). This reduction in parasite burden correlated with an increase of mouse IL-2 and IL-4, indicating that TAM can affect host physiology to protect against parasite invasion. At the same time, TAM appears to disrupt the estradiol-dependent process of parasite reproduction by binding to a parasite estrogen-receptor-like protein (62, 63). In hamsters, TAM prevented the establishment of intestinal infection by the adult worm by 70% (64). *In vitro* studies of TAM activity have also determined that the drug is cysticidal for both T. crassiceps and T. solium (62, 64). *In vitro*, TAM also inhibits parasite scolex evagination of T. solium cysticerci in a dose-dependent manner and completely prevented the differentiation from cysticercus to adult worm at 0.5 µM (64). There appears to be a consensus in the literature that the antitaeinal activity of TAM is linked to antiestrogen activity because estrogen is synthesized by the parasite and is linked to parasite reproduction.

The antiestrogen properties of TAM are also thought to contribute to the mechanism of its activity against Trypanosoma cruzi. Specifically, TAM has *in vitro* activity against T. cruzi amastigotes, epimastigotes, and trypomastigotes with EC_50_s ranging from 0.7 to 18 µM (65). *In vivo* experiments using Swiss male mice, Swiss female mice, and BALB/c mice found, however, that TAM was not effective in clearing infection or decreasing parasite load.

The antimalaria activity of TAM, CLM, and a TAM analog has also been investigated (66 – 69). *In vitro* studies in Plasmodium falciparum showed CLM inhibited parasite growth by 80% at 10 µM with IC_50_ values of 6 µM (69). In an *ex vivo* infection model using P. berghei and Huh-7 (human hepatoma) cell lines, TAM and CLM inhibited volume-regulated anion channels (VRAC) with IC_50_ values of 4 µM (67). In a red blood cell model, 10 µM TAM or 10 µM CLM inhibited parasite growth in a time-dependent manner and was more effective within the first 24 h than at 25 to 48 h. The time dependency indicates that mechanisms other than VRAC inhibition are likely to be operative (67).

TAM and CLM also inhibit intracellular development of malarial parasites in the liver during the first 48 h of infection, when drug is given prophylactically before introduction of sporozoites (67). In contrast, treatment at 24 h postinfection had less of an effect (67). An alternative biological target, the sphingolipid biosynthetic pathway, has also been proposed for the activity of TAM against the trophozoite and schizont stages of P. falciparum (66). Specifically, TAM inhibits the synthesis of glucosylceramide synthase, sphingomyelin synthase, and glycosylinositol phospholipid (66). Overall, the activity of TAM and CLM is quite low compared to other antimalarial drugs and is likely the reason that these molecules have not been developed further.

### Antibacterial activity.

Repurposing triphenylethylene molecules as antibacterials is a relatively new concept, most likely due to the fact that the pipeline for new antibiotics was reasonably robust until recently. Initial drug screens identifying such antibacterial activity were reported beginning in 2013 (70). This screen was for FDA-approved drugs that caused lysis in the so-called ESKAPE pathogens, Enterococcus faecium, Staphylococcus aureus, Klebsiella pneumoniae, Acinetobacter baumannii, Pseudomonas aeruginosa, and E. coli. TAM and CLM showed bacteriolytic effects against planktonic E. faecium and A. baumannii (70). TAM and CLM have an MIC of 8 µg/ml against E. faecium. In addition, TAM was active in an *in vivo* Galleria mellonella model of E. faecium infection where it prolonged survival in a dose-dependent manner compared to controls (70). In addition, the MIC of CLM against both S. aureus and Bacillus subtilis is 8 µg/ml (71).

TAM, CLM, 4-hydroxytamoxifen, and the non-triphenylethylene-based SERM RLX are active against M. tuberculosis. Against lab and clinical isolates of M. tuberculosis, TAM, 4-hydroxytamoxifen, and RLX are active at 3 to 20 µg/ml, 2.5 to 10 µg/ml, and 10 to 20 µg/ml, respectively (72 – 74). TAM also decreases the number of intracellular M. tuberculosis organisms in macrophages in a dose-dependent manner (74). TOR has been investigated as a potential treatment option for oral bacterial infections caused by P. gingivalis and S. mutans with MICs of 12.5 µM and 25 µM, respectively (75). TOR also inhibits bacterial growth and biofilm formation on titanium in a dose-dependent manner (75).

*In vitro* combination studies using TAM or 4-hydroxytamoxifen with the current antituberculosis drug rifampin, isoniazid, or ethambutol result in enhanced inhibition of M. tuberculosis growth (73, 74). *In vitro* fractional inhibitory concentration assays using CLM and clinical antibiotics against methicillin-resistant S. aureus identified many synergistic relationships with β-lactams and cephalosporins and additive interactions with other antibiotics. In addition, the combination of TAM with some of these drugs was able to restore susceptibility to resistant isolates (71). Additionally, *in vitro* combinations of TAM, TOR, or RLX with polymyxin B are synergistic against P. aeruginosa, K. pneumoniae, and A. baumannii growth in FIC assays and show increased activity in time-kill assays (76). Polymyxin B combined with TOR inhibits P. aeruginosa biofilm formation, damages and depolarizes the cytoplasmic membrane, and increases cellular reactive oxygen species (76).

The mechanism of the antibacterial activity of TAM has yet to be clearly defined. It has been reported to disrupt the membrane of nonpathogenic Bacillus stearothermophilus, resulting in leakage of cytosolic contents and cell death (77). This is consistent with the fact that TAM was identified in a screen using an assay for cell lysis (68). Additionally, TAM, 4-hydroxytamoxifen, and RLX act as ionic protonophore uncouplers that collapse the mitochondrial membrane potential of M. tuberculosis (72). Indeed, this effect has also been observed with the yeast Saccharomyces cerevisiae as well as other bacteria, suggesting it is an important aspect of its mechanism of action (73). CLM was also identified in a specific screen to identify inhibitors of bacterial cell wall biogenesis. Follow-up studies showed that CLM inhibits undecaprenyl diphosphate synthase in S. aureus (71). Finally, TOR also appears to interact and disrupt bacterial membranes. In P. gingivalis, TOR permeabilizes the membrane, causes membrane damage, and binds to LPS (75). *In* Francisella novicida, TOR has also been found to permeabilize cell membranes (78). Based on the work of Feng et al., TAM represents an example of a molecule that has effects on both membranes and specific protein targets that contribute to its overall antibacterial activity (72).

### Conclusion.

The anti-infective activity of molecules of the triphenylethylene class is quite broad, encompassing medically important bacteria, fungi, viruses, and parasites (Table 1). As such, it appears to be a biologically privileged scaffold. This broad spectrum of activity against microbes as well as a drug with activity against mammalian targets is likely related to its amphipathic chemical properties with a hydrophobic aromatic core linked to a basic amine function (30). Indeed, a tamoxifen analog lacking the amine function is completely inactive as an antifungal. These properties could imply that the molecules’ non-estrogen-related activities are simply nonspecific effects. However, the structure-activity studies that are available for triphenylethylenes indicate that changes in structure that are unlikely to affect their bulk properties have significant effects on their anti-infective activity (30). Thus, it seems that medicinal chemistry-based optimization of this pharmacologically attractive scaffold could lead to molecules with the right balance of activity and toxicity to be useful in the anti-infective space.

**TABLE 1:**
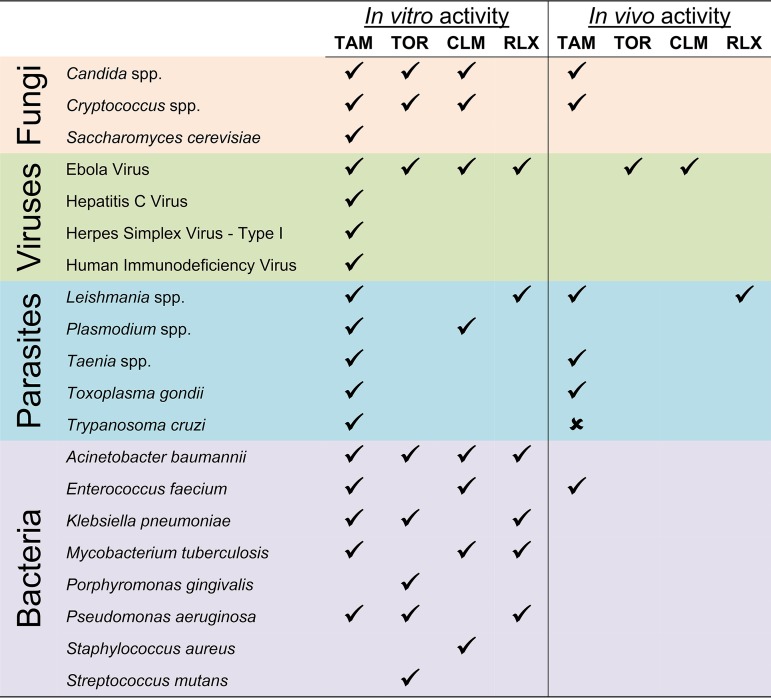
Tamoxifen, toremifene, clomiphene/clomifene, and raloxifene repurposing for infectious disease^a^

a TAM, tamoxifen; TOR, toremifene; CLM, clomifene; RLX, raloxifene. A checkmark denotes activity, an X indicates inactivity, and empty spaces indicate that no studies have been done.

The direct repurposing of tamoxifen appears to be the most promising. This is based on the fact that micromolar concentrations of the drug have been achieved in the context of its use as an adjuvant therapy for glioblastoma (79). Although these doses are approximately 10 times that used for breast cancer, patients tolerate the elevated concentrations well. The amphipathic nature of the drug also allows it to concentrate into tissues such as the brain quite well (80). As such, brain concentrations well above its MIC against C. neoformans are achievable. Accordingly, these considerations led to a phase II trial to test its activity as an adjuvant for cryptococcal meningitis (31). Similarly, the tolerability and distribution of TAM raise the possibility that it may be repurposed for some of the other infectious indications discussed above.
